# Analysis of microsatellite polymorphisms within the GLC1F locus in Japanese patients with normal tension glaucoma

**Published:** 2010-03-17

**Authors:** Kaori Murakami, Akira Meguro, Masao Ota, Tomoko Shiota, Naoko Nomura, Kenji Kashiwagi, Fumihiko Mabuchi, Hiroyuki Iijima, Kazuhide Kawase, Tetsuya Yamamoto, Makoto Nakamura, Akira Negi, Takeshi Sagara, Teruo Nishida, Masaru Inatani, Hidenobu Tanihara, Makoto Aihara, Makoto Araie, Takeo Fukuchi, Haruki Abe, Tomomi Higashide, Kazuhisa Sugiyama, Takashi Kanamoto, Yoshiaki Kiuchi, Aiko Iwase, Shigeaki Ohno, Hidetoshi Inoko, Nobuhisa Mizuki

**Affiliations:** 1 Department of Ophthalmology, Yokohama City University School of Medicine, Yokohama, Kanagawa, Japan; 2 Department of Legal Medicine, Shinshu University School of Medicine, Matsumoto, Nagano, Japan; 3 Department of Ophthalmology, University of Yamanashi, Faculty of Medicine, Yamanashi, Japan; 4 Department of Ophthalmology, Gifu University Graduate School of Medicine, Gifu, Japan; 5 Department of Surgery, Division of Ophthalmology, Kobe University Graduate School of Medicine, Kobe, Hyogo, Japan; 6 Department of Ophthalmology, Yamaguchi University Graduate School of Medicine, Ube, Yamaguchi, Japan; 7 Department of Ophthalmology and Visual Science, Graduate School of Medical Sciences, Kumamoto University, Kumamoto, Japan; 8 Department of Ophthalmology, University of Tokyo School of Medicine, Tokyo, Japan; 9 Division of Ophthalmology and Visual Science, Graduated School of Medical and Dental Sciences, Niigata University, Niigata, Japan; 10 Department of Ophthalmology and Visual Science, Kanazawa University Graduate School of Medical Science, Kanazawa, Ishikawa, Japan; 11 Department of Ophthalmology and Visual Science, Graduate School of Biomedical Sciences, Hiroshima University, Hiroshima, Japan; 12 Department of Ophthalmology, Tajimi Municipal Hospital, Tajimi, Gifu, Japan; 13 Department of Ocular Inflammation and Immunology, Hokkaido University Graduate School of Medicine, Sapporo, Japan; 14 Department of Genetic Information, Division of Molecular Life Science, Tokai University School of Medicine, Isehara, Kanagawa, Japan

## Abstract

**Purpose:**

To investigate whether the GLC1F locus is associated with normal tension glaucoma (NTG) in Japanese patients.

**Methods:**

We recruited 242 unrelated Japanese subjects, including, 141 NTG patients and 101 healthy controls. The patients exhibiting a comparatively early onset were selected as they suggest that genetic factors may show stronger involvement. Genotyping and assessment of allelic diversity was performed on 11 highly polymorphic microsatellite markers in and around the GLC1F locus.

**Results:**

Individuals carrying the 163 allele of D7S1277i had a statistically significant increased risk of NTG (p=0.0013, pc=0.016, OR=2.47, 95%CI=1.42–4.30). None of the other markers identified significant loci (pc>0.05) after Bonferroni’s correction.

**Conclusions:**

These findings suggested that the genes in the GLC1F locus may be associated with the pathogenesis of NTG.

## Introduction

Glaucoma causes permanent damage of the retina and optic nerve, leading to vision loss and blindness [1]. Primary open-angle glaucoma (POAG) is the most common type of glaucoma and normal tension glaucoma (NTG) is an important subset of POAG; while many POAG patients have high intraocular pressure (IOP) [2], NTG patients have statistically normal IOP [3-5]. NTG is more prevalent in the Asian population, in particular in the Japanese population [6-8]. The diagnosis of glaucoma is based on a combination of factors including optic nerve damage and specific field defects, with IOP being the only treatable risk factor. However, NTG is underdiagnosed: it usually presents late in life after loss of the visual field because it may be asymptomatic with normal IOP.

Glaucoma is recognized as a multi-factorial disorder [9]. Many genes are associated with glaucoma, and several are specifically involved in open-angle glaucoma [10-14]. The GLC1F locus on chromosome 7q35-q36 is the sixth gene locus for POAG [15].

Although NTG may be associated with the GLC1F locus, such an association has not been investigated. Thus, we performed microsatellite (MS) mapping around the GLC1F locus in Japanese NTG patients.

## Methods

### Subjects

We recruited 242 Japanese subjects from Yokohama City University, Yamanashi University, Gifu University, Kobe University, Yamaguchi University, Kumamoto University, Hokkaido University, Tokyo University, Niigata University, Kanazawa University, Hiroshima University, Tajimi Municipal Hospital, and Tokai University, all in Japan. Of these subjects, 141 had NTG, and 101 were control subjects. The criteria used for the diagnosis of NTG are previously described [16]. The mean age of the patients was 47.30±1.29 years old, and the male: female ratio was 0.92. The mean refraction value was −3.75±0.35 diopters (D), and the mean deviation observed in the Humphrey^®^ static visual field determination (Carl Zeiss Meditec, Oberkochen, Germany) was –10.19±0.94 dB. The age- and sex-matched controls were not affected by glaucoma or any local or systemic illnesses known to cause optic disc or visual field changes. The control cases had no myopia or had mild myopia with refractive errors of –3.00 D or less. Therefore, the groups were somewhat different mean refraction errors. All subjects had similar social background and resided in the same urban area, and provided informed consent. The study was conducted in accordance with the Declaration of Helsinki and subsequent revisions thereof.

### Analysis of the 11 microsatelllite loci

**Figure 1:**
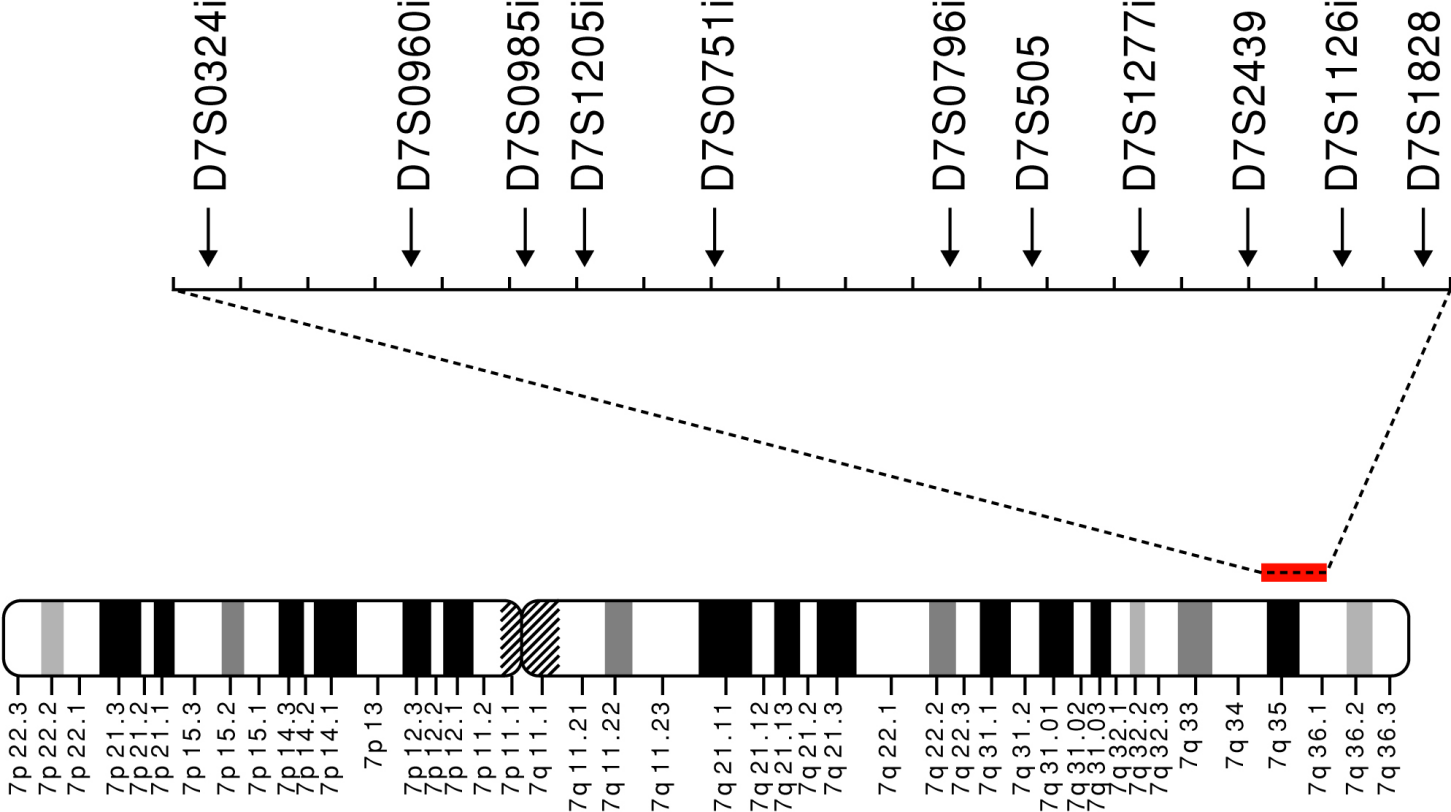
Location of the 11 microsatellite markers used in this study. All the markers were located on chromosome 7q35–36.

Genomic DNA was extracted from blood using the QIAamp DNA Blood Mini Kit (Qiagen, Hilden, Germany) or with the standard guanidine method. Eleven MS markers within the GLC1F locus were selected based on the National Center for Biotechnology Information (Figure 1). Polymerase chain reaction (PCR) was performed in a 12.5 µl reaction mixture comprising PCR buffer, genomic DNA, 0.2 mM dinucleotide triphosphates (dNTPs), 0.5 µM primers, and 0.35 U Taq polymerase. The PCR conditions were as follows: 94 °C for 5 min, followed by 30 cycles of denaturation at 94 °C for 30 s, annealing at 56 °C for 30 s, extension at 72 °C for 1 min, and a final elongation step at 72 °C for 10 min. The reaction was performed in a PCR thermal cycler (GeneAmp System 9700; Applied Biosystems, Foster City, CA). The forward primers were labeled at the 5′ ends with 6-FAM, 3-PET, 2-VIC, or 2-NED fluorescent dye (Sigma-Aldrich, St. Louis, Mo; Table 1). To determine the number of MS repeats, the PCR products were denatured at 97 °C for 2 min, mixed with formamide, and electrophoresed using an ABI3130 Genetic Analyzer (Applied Biosystems). The number of MS repeats was estimated by the Southern method with a GS500 TAMRA size marker (Applied Biosystems) and automated with GeneScan 672 software (Applied Biosystems).

**Table 1:** Primer sequences of 11 microsatellite markers used in this study.

**Locus**	**Dye**	**Orientation**	**Sequence (5′ to 3′)**
D7S0324i	FAM	F	TTTAACAGTTCACACCATGTAAATC
		R	TTCGAAGCACTACCAAGTCTAC
D7S0960i	PET	F	GCAATTGTATTCGTTTCATTAG
		R	CATTACAGGCGTGAGCTAC
D7S0985i	VIC	F	CTGGCTAACATGGTGAAAC
		R	GAGAACCTCAGTTGAGGTAGAG
D7S1205i	NED	F	TCTGCCTAGCACTAGAAAGAAG
		R	GGCTCTGTTTACTATACTGGAGG
D7S0751i	FAM	F	TGGCATAAGCTATTTGTATGTTTA
		R	TACAGTGAGCTATGATGGCAC
D7S0796i	NED	F	CGTATGGATACCTATGTAACAAAC
		R	TAATTAGACCACATTTAACCAGAC
D7S505	VIC	F	ACTGGCCTGGCAGAGTCT
		R	CAGCCATTCGAGAGGTGT
D7S1277i	PET	F	TGTCTTCTGAGACTGTAAGATGTTC
		R	TGGGAGAACAGTAGGATGG
D7S2439	FAM	F	CAGCAAAAGGTACAGCAATTTC
		R	AAAGTCTACGCCGCATTC
D7S1126i	FAM	F	CATGCTGAGCCTCAACTAC
		R	CTGTTGGACTCGTACTAAGATTAC
D7S1828	PET	F	TCTTTCCTTTCCTGCATCAC
		R	AGAATCTTGACATTATCTGACTTCA

### Statistical analysis

Allele and phenotype frequencies were estimated by direct counting. The allelic frequencies between the patients and controls were evaluated using Fisher’s exact test. The probability of association was corrected using Bonferroni’s inequality method. A corrected p (pc) value of <0.05 was considered statistically significant. Statistical analyses were performed with SPSS software (version 10.1; SPSS Science, Chicago, IL).

## Results

The proportion of allele frequencies and phenotype frequencies for the markers were consistent with Hardy–Weinberg equilibrium. Table 2 shows the allele frequencies of MS markers, with each allele designated by its amplification size. The frequency of the 163 allele of D7S1277i was higher in the patients than in the controls (p=0.0049, OR=1.97, 95%CI=1.22–3.17); however, after Bonferroni’s correction, the difference between the groups was insignificant (pc=0.063).

**Table 2:** Allele and phenotype frequencies of 11 microsatellite markers in NTG cases and controls

** **	** **	** **	**Frequency, n (%)**		** **	** **	** **
**Marker**	**Number of detected allele**	Allele*	**Cases (n=141)**	**Controls (n=101)**	**P**	**Pc**	**Odds ratio (95%CI)**
**Allele**							
D7S0324i	10	171	50 (17.7)	27 (13.4)	0.20		
D7S0960i	3	429	51 (18.1)	48 (23.8)	0.13		
D7S0985i	6	462	4 (1.4)	8 (4.0)	0.076		
D7S1205i	6	158	81 (28.7)	61 (30.2)	0.73		
D7S0751i	8	359	25 (8.9)	23 (11.4)	0.36		
D7S0796i	18	418	44 (15.6)	20 (9.9)	0.068		
D7S505	9	265	8 (2.8)	3 (1.5)	0.33		
D7S1277i	13	163	70 (24.8)	29 (14.4)	0.0049	0.063	1.97 (1.22–3.17)
D7S2439	13	210	28 (9.9)	11 (5.5)	0.074		
D7S1126i	9	276	123 (43.6)	99 (49.0)	0.24		
D7S1828	6	365	100 (35.5)	89 (44.1)	0.056	0.34	0.70 (0.48–1.00)
Phenotype**							
D7S1277i	13	163	65 (46.1)	26 (25.7)	0.0013	0.016	2.47 (1.42–4.30)
D7S1828	6	369	97 (68.8)	57 (56.4)	0.049	0.29	1.70 (1.00–2.89)

The asterisk indicates that alleles with >1% of frequency and found to have the most difference in allele frequency between cases and controls in each marker are shown. Each allele was named by the size of its amplification. The double asterisk indicates that only alleles with a p<0.05 are listed.

Table 2 also shows the phenotype frequencies of D7S1277i and D7S1828 with p<0.05. The 163 allele of D7S1277i and the 369 allele of D7S1828 were associated with the risk of NTG (p=0.0013, OR=2.47, 95%CI=1.42–4.30, p=0.049, OR=1.70, 95%CI=1.00–2.89, respectively). After Bonferroni’s correction, the frequency of D7S1277i 163 allele remained significantly different between the groups (pc=0.016).

## Discussion

The allele frequencies of GLC1F significantly differed between POAG patients and healthy controls in the American population [15]. The prevalence of POAG was unrelated to the GLC1F locus in a Finnish family, but the relationship between GLC1F and NTG was not studied [17]. We selected NTG patients with precise criteria because the existence of various clinical entities can lead to a lack of statistical validity in case-control analyses. With used 11 MS markers located in the GLC1F locus to detect an association between the GLC1F locus and NTG, and identified a markedly increased frequency of the D7S1277i 163 allele in patients, compared to controls (46.1% versus 25.7%).

Our data suggested that the gene(s) in the D7S1277i locus on 7q36.1 may be significantly associated with the risk of NTG. D7S1277i is located in the intron of the *AGAP3* (ArfGAP with GTPase domain, ankyrin repeat and PH domain 3; also known as *CRAG*) gene, a GTPase activating protein for ADP ribosylation factors. AGAP3 is a novel GTPase that accelerates the degradation of abnormally elongated polyglutamine proteins through intranuclear inclusion body formation. *AGAP3* is induced by reactive oxygen species and inhibits the progression of polyglutamine disease [18]. Although the association between *AGAP3* and glaucoma is unclear, *AGAP3* polymorphisms may act as risk factors in the development of NTG.

D7S1277i may be associated with other gene(s) but not *AGAP3* since MS markers are highly polymorphic and generally show wide linkage disequilibriums within 100–200 kb [19-24]. Twenty genes are located within the 200 kb region of D7S1277i. *NOS3* (nitric oxide synthase 3) is located ~100 kb centromeric of D7S1277i and has been reported as a POAG candidate gene [15]. NOS catalyzes the production of nitric oxide (NO). NO is involved in vasodilation and the regulation of ocular flow [25]. Ocular NO production may be important in the neuroprotection of retinal ganglion cells [26]. A *NOS3* polymorphism was significantly associated with glaucoma with migraine, but not with NTG or POAG in a case-control study [27]. Other *NOS3* polymorphisms also showed significant association with female POAG with high IOP in a case-control study [28]. Therefore, *NOS3* could affect the phenotype of glaucoma patients, and a disease phenotype-stratified analysis of *NOS3* in our NTG patients is required.

In conclusion, we performed an association analysis of the GLC1F locus using MS markers in NTG patients and detected an NTG-associated region in the GLC1F locus. Further studies in the region might help identify the pathogenic gene(s) of NTG.
